# Is Sport Sustainable?—It Depends!

**DOI:** 10.3389/fspor.2021.679762

**Published:** 2021-10-28

**Authors:** Jan Ove Tangen

**Affiliations:** Department of Sports, Physical Education and Outdoor Studies, Faculty of Humanities, Sports and Educational Science, University of South-Eastern Norway, Kongsberg, Norway

**Keywords:** sport, sustainability, sociological systems theory, civilizations rise and fall, new society

## Abstract

Is sport sustainable? Could sport be sustainable? Is there hope? The short answer is: it depends. This article discusses the six most essential issues on which answers to the questions above depend. First, it depends on what we already know about sport and sustainability. Second, it depends on how we observe or define sport and sustainability, respectively. Third, it depends on the ontology and epistemology on which the definitions and theories are based. Fourth, it depends on how we describe and explain the relationships and dependencies between sport, society and the environment. Fifth, it depends on how historical and sociological theories describe and explain how societies and civilizations operate, develop and eventually collapse. Sixth, it depends on whether we believe is it possible to plan and steer the future. These conditions indicate that the questions are challenging to answer but not impossible. Based on sociological systems theory, the author concludes that sport will not be sustainable unless modernity changes into a different kind of society—a world society that operates and governs from the binary code of sustainable/unsustainable instead of today's statal code of power/powerless.

## Introduction

In March 2019^1^, I was watching the television broadcast of the ski-jump contest at Holmenkollen Ski Festival. For years, this yearly festival has had a national significance, and the ski-jump facility has long been a symbol of national identity and pride. Suddenly, looking at the spectacular performances, I noticed that the bib numbers' sponsor pinned to ski jumpers' chests were CHOOSE^2^, an international actor in the business of selling carbon compensations. I become shocked and disappointed. Is this how the ski sport will reduce their carbon emission problem? Will all sports follow suit, buying carbon offsets so athletes and supporters can travel with a clean conscience while leaving developing countries responsible for producing energy from clean and renewable sources? To me, this is almost like cheating or paying indulgence. Sport, I thought, as all other organizations and institutions in modernity, should endeavor to choose the right and most effective means to minimize their impact on environmental, economic and social sustainability. Is modern sport willing to go all the way to be sustainable? Is it possible at all for modern sport to be sustainable? Is there hope? The last three questions are crucial to answer.

I started reading academic articles and books realized soon that the answer was: “It depends!” This article will address what I saw as the six most essential issues that answer the three questions above. The answers depend on what we already know, what the research on this topic has revealed, and whether the right questions have been asked. Second, I discovered that the answers depend on how both sport and sustainability were defined and delimited in public discourses and current research. Using one definition of sport combined with one definition of sustainability led both commentators, debaters and researchers to very different conclusions about whether sport can be sustainable than if they used other definitions. Third, it also struck me that the answers depend on the ontology and epistemology embedded in the definitions, analysis and scientific theories. Taking the world as it is, seemed natural to some debaters and researchers, while others insisted that everything is socially constructed. The different ontologies and epistemologies affected, of course, which conclusions the debaters and researchers could draw on the question above. Fourth, and most importantly, ontologies and epistemologies also influence how researchers think about how societies work, how they operate and develop, and how this may impact individuals in particular and nature in general. If one assumes that sport is produced, maintained and developed within a society, then whether the sport is sustainable depends on and is influenced by its attributes and operations. Fifth, my reading revealed a need for theories that represented the complex and intricate relationships between individuals, sport, society, and the environment, or at least discuss the rise and fall of civilizations, including modern society's prospects. Sixth, whether the sport is sustainable depends essentially on whether sport is able to steer itself toward sustainability or whether there is a possibility of political steering within and between societies over centuries, and not least, steering for a certain future. In the following, I will delve into these six issues and show how they frame the answers I am able to give to the questions above.

The current literature seldom addresses these framing and determining conditions for our understanding. That is understandable in light of the vast complexity and intricacy of the issues. Nevertheless, this article will tentatively address these six necessary conditions for answering whether the sport is, or can be, be sustainable. I think the time is ripe to lift these issues forward and discuss them. I do not pretend to give neither a comprehensible nor final answer to the question, “Is sport sustainable?” I only want to raise awareness about how our thinking is conditioned and determined. In other words, I suggest a theoretical perspective to understand the relationship between sport and sustainability and eventually help the sport to become sustainable.

In the following, I first present and discuss some of the most definitive studies on sport and sustainability. Then I invite the reader to reflect upon how we as ordinary humans and as researchers observe the world and find ways to describe it. From this detour in the philosophy of science, I look into how “sport” could be defined and delimited and which consequences these efforts have on the question of “is sport sustainable?” Next, I do the same definitional exercise concerning “sustainability,” showing some of the attempts to characterize this phenomenon and document the complexity and intricacy the definitions reveal. Doing so gives me the tools to discuss how sport influences and cope with sustainability and concludes that sport seems both unwilling and unable to reduce its impact on sustainability. This conclusion leads me to claim that sport, metaphorically speaking, carries the DNA of modernity and cannot be more sustainable nn modern society. This claim again opens for a discussion of relevant theories to explain how societies and civilizations develop and fall, how they react to challenges and catastrophes, how they change and restructure. Therefore, the question of whether the sport is sustainable has to be placed in that historical context. I will therefore look into some “grand theories” for an answer, theories that address the rise and fall of societies and civilizations and why some collapse and others survive (Diamond, 2003, 2005; Turchin, 2007). Since the pressing question of whether modern sport can be sustainable, I will also relate sport and sustainability to the challenges facing the 21st century (Harari, 2018). Most important, however, is my use of the sociological theory of Niklas Luhmann (Luhmann, 1984, 1997a). In my opinion, his approach offers the most convincing perspective on how societies have evolved historically and may develop in the future. In particular, his view on modernity and its attributes (Luhmann, 1998) helps me answer whether sport and society can be sustainable.

This article is an explorative and tentative theoretical discussion of the issues raised above. The space limit allows only painting a broad and impressionistic picture, using stiff brushes and intense colors. This contribution is, therefore, more of an essay than a traditional scientific article. In this particular thematic context, I find it very appropriate to follow Bateson's (1972) advice of “connecting the dots” rather than “analyzing the dots.” This task is more urgent now than ever before, in particular on the topic of sustainability. As Fritjof Capra wrote: “… this [connecting the dots] is critical today not only in science but also in politics and civic life, as most of our political and corporate leaders show a striking inability to connect the dots.”^3^

## Sport and Sustainability—Attentions and Prerequisites

What do we know about sport and sustainability? A Google search using the keywords “sport and sustainability,” returned almost 230 million hits. Searching on Google Scholar with the same keywords gave nearly 560,000 hits. Both findings indicate the relatively considerable *attention* focused on this topic as well as implying its importance. Searching for each keyword alone came up with one surprising result: “Sport” gave 10.5 billion Google hits and “sustainability” 0.8 billion. The great interest in sport indicates that it has considerable potential to impact the public's observations and responses to the demands of being more sustainable. This potential is one crucial reason for making an in-depth analysis of sport and sustainability.

This rather crude and superficial search showed that “sport and sustainability” is an *important* topic worldwide, at least on the Internet. Second, it documented that a comprehensive review of all aspects of “sport and sustainability” is too large of a task for one scholar to cover in one article. Third, I got a provisional impression of the variety and debt of the postings and papers on this topic to position my contribution in the societal and scientific context.

Looking through a fraction of the links popping up during the Google search, I discovered that sport is *doing* a lot to be more sustainable. Webpages and articles tell us that sport is concerned with pollution, recycling, carbon emissions, and so forth. The results inform us that many voluntary and organizational initiatives are trying to make the sport more sustainable, such as banning the fossil fuel industry from sponsoring sports and committing sports organizations to adopt a climate action framework. We learn also of conferences that have stated that only exact, bold actions will position sport as a sustainability leader. We read that sustainability is one of the three pillars of the Olympic Agenda 2020. These searches indicate that sport and sustainability involve individuals, organizations, institutions, politics, economy, technology, and nature. They all seem to be entangled and dependent on and influenced by the actions and responses of others.

Most of the same issues and themes appeared in the scientific articles and books revealed by the Google Scholar search. As one can imagine, it is impossible to give credit to all the 560,000 hits. Many of these articles focus on how sports organizations in general and the sports industry, in particular, have developed *strategies* to address relevant issues. The concern for sustainable management of sport has triggered two types of environmental initiatives: to reduce the harmful carbon footprint and to use sports to raise ecological awareness (Trendafilova et al., 2014). Sports organizations around the world have adopted many environmental programs. My concern with this adoption is the lack of a thorough theoretical analysis of what makes sport unsustainable in the first place. To put it bluntly: it is not sufficient to recycle cups and bottles after a soccer match, heating the stadiums with renewable energy, more use of public transportation by subways, etc. These actions are more or less symptomatic treatments. One has to address more precisely why fans, facilities, transportations etc., have emerged at all. “Something” has transformed sport from a ludic, informal, leisure activity to a global, commercialized, professionalized spectacle with grave consequences for environmental, economic, social and psychological sustainability. It is crucial to identify this “something” to solve the challenge of sustainability.

I In the following, I will focus only on exemplary scientific contributions that outline the prerequisites to make sport sustainable or not. For example, Lindsey (2008) claims that sustainability is a crucial issue in sports development these days. Yet, he also claims that research on sport and sustainability lacks a *theoretical* underpinning. Lindsey's contribution in that regard is to conceptualize sustainability in sport development. I fully agree with Lindsey that the research needs more theoretical support. However, I am surprised that Lindsey only addresses the conceptual problem of sustainability. Why didn't he also discuss the conceptualization of sport? Without taking a closer look at the sport's distinctiveness and operations that have consequences for sustainability, much of the theoretical efforts may prove worthless. And is “sport development” the proper perspective to make the sport more sustainable?

*Socialization* and *learning* are prerequisites for convincing individuals and groups to become more sustainable. McCullough et al. (2016) give an extensive review of the current research on environmental sustainability in sport, focusing on sports organizations. They further suggest a model of how individuals, organizations, and institutions may learn and act to implement waves of environmental sustainability into the sport. The authors also offer a conceptual framework and empirical descriptions to understand how the awareness and importance of sustainability ebb and flow in sport and why this happens. Undoubtedly, this is essential knowledge about how sports organizations respond to the sustainability issue. However, they do not have an explicit discussion of the autonomy of sport that may obstruct parts of the efforts to be sustainable or discuss what aspect or dimensions of environmental sustainability they are studying. In particular, a discussion about whether sport in its modern configuration could be sustainable at all, could have been interesting.

The comments above lead to a discussion of whether the ambition of winning could be combined with the responsibility of taking care of the environment. Barker et al. (2014) touch on this question in their article “High-performance sport and sustainability: a *contradiction* of terms?” To a certain degree, they show that elite sport and sustainability are incompatible and contradictory. The authors point out that many athletes exploit their bodies for short-term gain or cheating for victory. Elite athletes face extreme pressure. The selection process among capable athletes, the continual stress to perform, and the desire for success almost force athletes to do whatever it takes to be the best. Even if the authors suggest that it is difficult to see how elite sport could be sustainable today, they nevertheless express hope if elite sport pays more attention to educating the athletes on sustainability. As they claim, “Learning is essential” (p. 7). I agree that learning is essential in all human practices. However, what is the main lesson athletes learn in sport? Is it to be fair and morally responsible, to be sustainability-conscious or a winner?

The Norwegian sports philosopher Sigmund Loland believes it is possible to be all three (Loland, 2006). This is a recurring theme in his extensive and inspiring writings. Loland believes that sport could be sustainable, given some *necessary changes*. In this article from 2006, he discusses whether the Olympic Games can serve their environmental concern for sustainable development by selecting and reforming individual sports. He answers his question by first defining the ideal of sustainable development and then reviewing Olympic sports, calling for reforms concerning the objective of sustainable development. Without going into detail about his rather convincing argumentation, I will focus on his reformist requirements. He argues, and I quote: “If we are to take the Olympic ambition of sustainable development seriously, not only record sports but sports with specialized performance ideals ought to be abandoned or reformed” (p. 150). This suggestion is somewhat radical. I find Loland's argumentation and conclusion interesting and very similar to my own. However, in my opinion, he does not quite settle the question. Specialization and breaking records are, metaphorically speaking, only elements and sequences of elements of Sport's DNA (more on this later). Loland does not identify “the genetic code” that orders such elements (records) and sequences of elements (specialization) in sport.

This short review brings forth two critical issues. First, these articles suggest necessary *prerequisites* for the sport to be, or become, sustainable. Second, the public and scholarly discussion seem to disregard, or at least pay little attention to, the *distinctiveness* and *operations* of sport as a social system and how these aspects work for or against the idea of sustainability. Both topics are decisive to reflect upon if we aim to answer whether the sport is, or can be, sustainable. More importantly, these topics are decisive to answer the vital question “Is there hope?” So, how can we identify both the prerequisites, distinctiveness and operations of sport as a social system and the traits of sustainability? To answer these questions, we first enter the playing field of ontology and epistemology. This detour is essential to discuss how we observe the appearance and reality of these phenomena and their reciprocal impacts.

## Observing the World—Some Epistemological Statements

As scientists, we ask a significant number of essential or ontological questions. “What is sport?” or “What is sustainability?” are two examples. Quite a few answers have been suggested on these two concepts. More important, one question often slips away: “How do we know?” We should remind ourselves of this epistemological question from time to time. Luhmann gives an answer I find compelling. As scholars, we should take “the world as it appears without asking ontological or metaphysical questions … Whatever appears can be interpreted as being the exclusion of other possibilities … If something appears as structure …, it is a strong argument for its being an indication of reality” (Luhmann, 1990a; p. 83). In other words, Luhmann is more concerned about epistemology than ontology, and so am I. *Identifying* the structure and operations of sport indicates its reality. Sports involves structure–fitness, music, and dance as well. As individuals, we observe the structured movements of fitness, dance, music, and sports. We take these structures for granted as an indication of reality and may choose to participate. Researchers take such structured activities as real and as expressions of a social institution's or a social system's existence, in my terminology. From this, researchers such as myself specify and explain how and why social systems emerge, how they are produced and reproduced.

Maturana proclaimed in the 1970s: “Everything said is said *by* an observer.” An intriguing answer to the critical question of “how do we know?” At a later conference, von Foerster cited and reformulated Maturana's postulate: “Everything said, is said *to* an observer” (von Foerster, 1979; p. 5). In doing so, von Foerster established a vital connection among three fundamental factors: an observer capable of formulating descriptions, a language connecting two observers, and the society that emerges when at least two observers use language. von Foerster also emphasized that every observer has cognitive blind spots. What we use to observe—be it microscopes or telescopes, concepts or theories—makes us see some things and disregard others. These ways of observing defines how we understand the “realities,” how we communicate about them, and how we deal with them. Realities as sports and sustainability are no exceptions.

Since I am the observer in this article, I have to clarify my *background* and *perspective* for observing sport and sustainability. I base my answer on 40 years of studies on sport as a social system, or more precisely, “How is sport possible?” (Tangen, 2004). My Ph.D. study started in 1982 with a Weberian approach to understanding the development of sport in the Western world. It ended up being a homage to Niklas Luhmann (Tangen, 1997). Since then, I have observed different aspects of sport and its psychological, social and environmental environment through this remarkable theory. I find the approach particularly suitable, supplemented with similar theories and concepts, for determining whether the sport is sustainable. In other words, I perform what Luhmann (1984) calls a second-order observation, observing other scholars' observations.

Luhmann (1984) developed Maturana's and von Foerster's epistemological point of departure further. His analysis of society is grounded on two *premises* that stem from the concept of observation. “(1) Every description of society must take place within society; that is, it is subject to observation, and this observation, at least today, is reflective. (2) Every description is bound to the basic structure of the operation of observing and therefore not overcome the limitations this implies.” (Luhmann, 1998; p. 78). These premises apply to everything I say in this article.

When observing, we make *distinctions*. We observe differences between this and that, focusing on the foreground and ignoring the background. For example, one crucial difference is whether the tennis ball is “in” or “out.” If the ball is in, one of the players earns points. The distinction between winner and loser is made when one of the players reaches a specific number of points. In this article, I use the distinctions sport/society, society/environment, problem/solution, and sustainable/unsustainable to discuss some crucial aspects of the driving question—“is sport sustainable?” The definitions and theories I present in the following are also distinctions, though very sophisticated distinctions.

Observing through differences is probably the most basic operation used to gain knowledge in biological, psychological, and social systems (Luhmann, 1984). Luhmann refers to the mathematician George Spencer-Brown's terminology that two operations are going on: *distinction* and *indication*. A distinction, or difference, is made between something and something else. The inside of something is then indicated or marked. When two or more individuals agree to determine who runs fastest, the social system of the sport emerge. A distinction between sport and everything else is drawn. The selection of athletes, the length of the run and the start and finish lines indicate that the system's inside is marked. The first athlete to reach the finish line is the fastest and, therefore, the winner (Tangen, 1997).

We use distinctions to describe the world and ourselves, making sense and gaining knowledge, be it sport, sustainability, science and other phenomena. Biological, psychic and social systems emerge from the *initial* drawing of a distinction between the system and the environment (Luhmann, 1984). Everything but the system itself is the environment. The individual psychological and organic system, other social subsystems, and nature are the sport system's environment. Identity is created when the difference between the system and the environment re-enters the system. References to and descriptions of itself and the environment express the system's knowledge about itself (self-reference) and the environment (external reference). This knowledge is practical and useful, but only temporary. The knowledge renders the system capable of maintaining and changing as responses to inputs from the environment. However, when the distinction between the system and the environment for some reasons ceases to be drawn, the system and the environment cease to exist.

From the initial distinction between the system and the environment, the system maintains and develops itself by making new distinctions. All social systems are *autopoietic*, which means that they produce, reproduce and organize all their constituent elements. They are self-creating, self-organizing, self-maintaining and self-developing entities. Nothing is imported “readymade.” They are operationally closed, which means that they operate only within themselves (Luhmann, 1984). The sport system produces its participants, performances, events, facilities, and rules (Tangen, 1997). However, the sport system continually observes and relates to the other subsystems of society. Each system decides its contact with its environment. It shields itself from countless influences while selecting a few relationships. Luhmann conceptualizes such relationships as structural coupling, a mechanism for mutual influence (Luhmann, 1984).

In other words, the social system of sport *observes* itself and the environment and gains *identity* as different from the environment and *knowledge* about its various impacts on the environment. It is *autopoietic*; it produces and reproduces everything it consists of. But what are these distinctions and indications that are operative in sport day in and day out? Which consequences for sustainability follows from the distinctiveness of sport and its autopoietic operations? I attempt to answer these questions in the following section.

## The Distinctiveness and Operations of Modern Sport

I think most of us will agree that modern sports are about winning and losing, following specific rules and norms, performed in distinct arenas and stadiums after training and preparing for months and years. Participants are included based on membership and selected to teams and contests according to specific performances and standards. The competitions, i.e., the matches, the downhill races, the tournaments, the marathons and so forth are events, scheduled and planned for months and years ahead. The events can be small as a soccer match for children or mega, as the Olympic Games. New playing fields and arenas are planned and built for specific purposes and different sports. Athletes, sports leaders, spectators and tourists travel between cities, countries and continents to compete, organize, support and experience the sport. These structures and operations are clearly observable, but what are the fundamental distinctions and indications behind them? Can a non-essential definition capture the more or less concealed distinctiveness of sports and sporting behavior?

I have observed that many social scientists do not define the social phenomena we call sport (Tangen, 1985, 1997). At best, some use ad *hoc* definitions that encompass somewhat different social phenomena as sport, fitness, exercise, physical education and so forth. The ad *hoc* definitions are presented in a rather superficial manner and passing. As mentioned earlier, I am also somewhat surprised to see that scholars put great efforts into defining sustainability but leave out their view on what is distinctive about sport and its operations. The absence of reflections on modern sport's distinctiveness and operations makes it difficult to conclude whether it is sustainable.

Roland Barthes once asked: “What is sport?” He continued: “Sport answers this question by another question: who is best?” (Barthes, 1961/2007; p. 58). From my perspective, he is right. It seems that every society, every culture, both historically and contemporary, had some body-based practices that could be intuitively observed as a sport. In contrast, other practices were intuitively considered as “something else.” In my research, I extracted some distinctive characteristics based on Luhmann's concepts elements, operations, and code. I ended up claiming that sport is “displaying and comparing bodily movements to determine who is the ablest, i.e., the winner” (Tangen, 1985, 1997, 2000). Sport is about winning and avoid losing. This distinction differentiates sport from other body-based practices like exercise, fitness, physical education, and so forth and differentiates sport from politics, science, economy, and religion (Schimank, 1988; Luhmann, 1990b; Stichweh, 1990).

Using Luhmann's terms, I claim that the binary and *primary* code of sport is “win/lose.” Other body-based practices such as exercise, fitness, and outdoor life activities have their code, which I do not have space to discuss. Everything going on in sport is about deciding who runs fastest, jumps highest, or is strongest. This code is observable in the epic story of Gilgamesh, in the description of the wrestling match between the great ancient Babylonian hero Gilgamesh and his friend Enkidu. We can observe the code in the “Iliad” when of funeral games of Patroklos during the siege of Troy is described. More so in the stories of the ancient Olympic Games in Greece, the cruel “Ludi” at the Roman amphitheater, the horse races at Circus Maximus as well as in the “leikir” of the Vikings, the tournaments of the medieval knights, the deadly ball games of the Mayans, and the Tikopian dart matches. They were all about winning and losing (Tangen, 1997).

However, the *primary* win/lose code was supplemented or extended during the 19th and 20th centuries. The sport system started to experiment with the *secondary* code of “improve/decline” to secure the victory (Tangen, 1997). It was not enough to be the best, but the athletes also had to be better, run faster, jump higher, and be stronger. Selecting the proper operations and avoid the improper ones concerning winning were easier using the secondary code. Whenever a loss was experienced, the reflections centered around possible reasons for the less successful performance. This change of focus opened up more possibilities but also more risks. The attempts to improve could fail. The contingency increased. However, the benefits were more promising than drawbacks. This *double*-code is the “driving force” behind all sporting practices, even though the “force” is much stronger in elite sport than leisure sport.

The “curse” of modern sport is that it is not enough to be the best among your peers. The nagging question continually “haunts” the participants: “Am I better than others in this local community, in the county, in my country, in the World?” This urge for *sporting omnipotence* had to be satisfied, virtually at all costs, with possible consequences for environmental, economic and social sustainability. The emergence of elite sport is a manifestation of this urge. Many contests are staged locally, nationally and internationally to determine who is best in running, jumping, throwing, swimming, playing soccer, and so forth. Even children and sporting amateurs find meaning in traveling around to compete, performing leisure sports. Parents or voluntary adults fill up cars with children to play soccer matches against a local city team. Amateurs travel from Norway to Italy to participate in Marcia Longa, a cross country ski race. Significant events, like World Cups, Olympic Games and other mega-events, attract spectators by the thousands, also traveling from home to the hosting city. They cheer on their national heroes and confirm the significance of sport in modern society. In particular, they applaud record-breaking performances, i.e., performances that exceed every other performance in the actual discipline. Athletes and amateurs travel to areas where they can exercise at high altitude to perform better at lower altitudes. Some escape winter and snow, travel to warmer places to play golf in winter times. Others travel to exotic locations to find the more giant and more exciting waves to surf on. Supporters, journalists, TV-camera-operators follow the tracks of the athletes and teams worldwide, leaving thousands of tons of carbon emissions behind them. All this traveling related to elite and leisure sport, is really alarming and should be analyzed and discussed in relation to sustainability.

Allen Guttmann's seminal work *From Ritual to Record* indirectly documents the development of sport's double-code when he describes sport's evolution from ancient Olympic sport to modern Sport (Guttmann, 1978). According to Guttmann, seven changes took place in modernity: secularization, equality, specialization, rationalization, bureaucratization, quantification, and the quest for records. Every one of these modern characteristics indicates the urge to improve and enhance the performances. Other scholars have added similar and supplementary features (Schimank, 1988; Stichweh, 1990). What is important to stress is that Guttmann's and others' characteristics imply that the double code of modern sport—the win/lose and improve/decline—is distinctive and operative in modernity. To enhance the performances and to win, every stone is turned. Only the sky is the limit. The Olympic motto “Citius–Altius–Fortius” manifests the distinctiveness and indicative operations in modern sports. The slogan also blatantly signifies the hyperbolic tendency of growth in modernity.

We have to realize that the double-code and modern sport motto are *pathological* with *pathogenic* consequences (Tangen, 1988, 2003). Athletes cannot run in fewer than zero seconds. Athletes will not high jump 6 meters without a pole. Athletes will not lift 5 tons in a bench press. Despite this, modern sport continues to improve as if these performances were possible. Such pathological ambitions demand vast resources and competencies. Modern sport has to cooperate with other social systems to get these resources and competencies. The social system of sport gives mass media access to events, contests, and profiled athletes in exchange for exposure and public attention. The sport system enters into contracts with sponsors in exchange for equipment and money. Modern Sport approaches governments and municipalities to get political support in exchange for grants to build facilities and training centers. The sport even places itself at the disposal as a laboratory for human enhancements and its athletes as “guinea pigs” in exchange for scientific knowledge and expertise (Hoberman, 1992) to realize the pathological ambition of *Citius–Altius–Fortius*. Modern elite sport is dangerous and endangered due to its double-code (Schimank, 2005).

In modern elite sports, each athlete, team, and nation search for a competitive edge (Pielke, 2016). The urge for sporting omnipotence has become an “arms race” made possible by the attention, resources, competencies, and legitimation from the other social subsystems just mentioned. This hyperbolic development has resulted in unanticipated but alarming internal and external consequences for sustainability. Besides, we should also pay attention to the impact of leisure sport on sustainability given the number of participants, their conspicuous consumption of sports related products and extensive traveling. However, the development of modern elite and leisure sport is not sufficient analyzed before we take a closer look at the intricate and complex relations between sport, society and sustainability.

## Sport, Sustainability, Societies and Civilizations

Sport is an essential element in modern society. If is affected by the society that surrounds it as well as affecting the society itself. Sustainability have impact on both society and sport and vice versa. To understand the complex entanglement between sport, society and sustainability, we also need to see this complex in light of the development of civilizations. Let's start with the claim that sustainability is not a new problem.

Sustainability has been observed and tried counteracted before. Studies of civilizations indicate that civilizations embed seeds to their own demise. Historically, a particular society's increased regional success was often followed by crises that were either resolved, thereby producing sustainability, or not, thereby leading to deterioration (Diamond, 2003; Turchin, 2007). However, the problem has accelerated in modernity as a consequence of this particular type of society. Now, the whole living Earth is threatened. Still, there is no consensus about how this problem should be conceptualized or solved (Purvis et al., 2019). However, scholarly observations and descriptions are essential to lifting forward and discuss in this context.

Carson's book *The Silent Spring* Carson (1962) exposed the pesticide DDT hazards and questioning the faith in technological progress. The book initiated a shift in global environmental consciousness. In 1972 the report *The Limits to Growth* (Meadows et al., 1972) was published. Using data modeling and simulation, the authors tried to calculate how long it would take before human consumption (of energy) exceeded the Earth's finite supply of resources. Both books emphasized the dangers of unlimited growth and progress, indicating that the environment has a homeostatic balance that should not be disturbed.

The Brundtland Report for the World Commission on Environment and Development introduced the term “sustainable development” (Imperatives, 1987) based on the idea of balanced development. The report emphasizes that sustainable development is a “… development that meets the needs of the present without compromising the ability of future generations to meet their own needs” (Imperatives, 1987; p. 16). The report further argues that sustainable development is “the process of people maintaining change in a balanced environment, in which the exploitation of resources, the direction of investments, the orientation of technological development and institutional change are all in harmony and enhance both current and future potential to meet human needs and aspirations.”

Since then, the debates have been intense about how the concept should be defined and how sustainability should be realized. Here it suffices to give some examples to indicate the difficulties of defining and delimiting this concept. The 2005 World Summit on Social Development identified three *pillars*, or domains, of sustainability: economic development, social development, and environmental protection. Three intersecting circles often represent these three pillars with overall sustainability at the center (Purvis et al., 2019). James and Magee (2016) suggested four *domains*: economical, ecological, political, and cultural sustainability. Another model (Thomas, 2016) indicates that humans attempt to achieve their needs and aspirations via *seven modalities*: economy, community, occupational groups, government, environment, culture, and physiology.

Lately, the UN has proposed 17 sustainable development *goals* to promote prosperity while protecting the planet. Each goal is substantiated by facts and concretized as actions. For example, goal 13, “Climate action,” states that “global emissions of carbon dioxide (CO_2_) have increased 50% since 1990.” Therefore, the UN calls us to take action “Act now to stop global warming.” In discussions of whether the sport system is sustainable, these goals are useful. I will later relate the sport system's distinctiveness and operations to some of these goals in particular.

However, one should also distinguish between *internal* and *external* sustainability. By internal sustainability, I mean how the distinctiveness, self-reflectiveness, and operations of the social subsystems affect the subsystems' inner states. For example, could the modern sport, in the long run, cope with the internal consequences of following the pathological code of continuous and limitless performance enhancement? External sustainability refers to how modern subsystems influence the environment, particularly some of UN's sustainability goals, such as individual health, equality, climate change and so forth. Traveling to national and international events and competitions is an example of how sport affects external sustainability. Could the modern globalized sport continue under the horizon of carbon offset and global warming due to traveling?

The discussion of sustainability and the conditions for it has to consider *theories of societies*, theories that observe societies' and civilizations' development and demise, and why some societies flourish and others founder. This article is not the place to delve into and discuss the benefits and shortcomings of the many lengthy and insightful books other scholars have written about this. I only want to mention the most relevant and exciting contributions as a backdrop for discussing sustainability and sport. Diamond's *Guns, Germs, and Steel* Diamond (2003) is a monumental study of the interaction between history, culture, climate and the environment. However, it is also a remarkable study of why Western civilizations came to dominate the world. Modern sports as athletics, basketball, soccer, baseball, cricket etc., played their part in Western societies' cultural imperialism (Guttmann, 1994). Diamond's follow up study, *Collapse*, explains why civilizations decline and demise (Diamond, 2005). Five factors that the civilizations often are responsible for themselves are operative: environmental *damage* like deforestation, pollution, soil depletion or erosion; *climate* change; *hostile* neighbors; *withdrawal* of support from friendly neighbors; and how a society or civilization *respond* to its problem, environmentally, politically or socially.

Turchin (2007) offers a supplementary explanation. In his book *War and Peace and War*, Turchin raises the question, “How are empires possible?” He focuses on essential aspects of how certain groups develop through time, not human individuals. Some groups develop and grow and become empires, mostly due to group traits and responses to events in the surrounding environment. Turchin claims that historical *dynamics* are a result of conflict and competition between groups. He also assumes that different groups have different degrees of cooperation among their members, resulting in various cohesiveness and solidarity. This latter aspect is coined *asabyia*, the capacity of a social group for concerted collective action. Groups of people, finding themselves on fault-line frontiers, develop a high capacity for collective action, enabling them to build empires. However, strong empires contain the seed of future chaos. Prosperity cause population increase and overpopulation and reduced fortunes for the elites. Discontent and strife emerge. Civil wars and popular rebellions burst out. Turchin also claims that rise and fall repeat in phases or cycles lasting 2 to 300 years. Our modern civilization is at the start of the decline if I understand Turchin correctly. The crucial question for modernity is: is there hope for stopping the fall?

Harari (2018) poses *21 Lessons for the 21st Century*, emerging from five challenges related to technology, politics, despair and hope, truth and adaptivity. Sustainability is only one part of one of the political challenges that face modernity, namely “Nationalism.” Harari claims that nuclear war, ecological collapse, and technological upheaval threatens human civilization's future. Somewhat ironic, he lifts the Olympic movement forward as an example of how nations can cooperate globally. He sees this competition between nations as an amazing geopolitical agreement. However, he does not discuss the possibility that the contract may result from one international organization's iron grip on the sport, an organization being criticized for being undemocratic, elitist, corrupt, and unsustainable. Is Harary implying that this form of government is needed to solve nationalism and the other 20 lessons with sustainability consequences? This issue has to be discussed.

The few characteristics, structures, processes and prospects presented above indicate the vast *complexity* and *entanglement* of sustainability, making it very difficult to determine whether the sport is sustainable. On the other hand, as a researcher, one cannot focus on only one or a few attributes, especially since many sustainability elements interact, depend on, and influence each other. This dilemma has no solution but being explicit on which sustainable dimensions, pillars or goals one focuses. In the following, I try to follow that principle. But again, my description is broad and sweeping. What I have tried to document is that the sustainability problem could not be solved without great changes in the society in particular, and the modern civilization in general. Hopefully, my analysis may be considered thought-provoking, but relevant.

Having taken these epistemological, theoretical and definitional detours, I am now ready to suggest a tentative answer to the questions, “Is sport sustainable?” and “Is there hope?”

## Sport and Sustainability

Modern sport's pathological ambition of breaking all records and transgressing all limits threatens its *internal* sustainability. The combined logic of winning and improving drives the sport system to produce better performances, more abled athletes, more systematic training, better equipment, more accommodating facilities, more efficient organizations, more spectacular events and more effective search for the best talents. This ambition already has severe effects on the sport itself and several of the UN's sustainability goals.

The boundless ambition of sport seems to carry its demise. Schimank (2005) pointed out how modern sport's autonomy is dangerous and endangered. It threatens the health of the athletes and the legitimacy of sport in society. These threats may reduce the willingness to participate and the support from governments and sponsors. As mentioned above, the Norwegian philosopher Sigmund Loland warns against Olympic athletes' specialization and record-breaking tendency (Loland, 2006). He claims that these tendencies have to be abandoned or reformed significantly. I think reforms are not enough. I am not sure that abandoning the tendencies are possible either. The modern urge to be sporting omnipotent is very strong. Put differently, the logic of sport threatens the sport itself, i.e., its internal sustainability. Besides, modern sports evoke excitement and patriotism, making it challenging to get rational arguments through in public discussions. Emotions trump reasons.

There seems to be a consensus about the benefits of sport on individual health, integration and equality, community feeling, learning of fair play, engaging in voluntary public engagement and so forth. However, these benefits must be compared and weighed against the disadvantages that sport brings about. Many athletes suffer from injuries, eating disorders, and lower self-esteem due to the urge to enhance their performances. Some athletes use legal and illegal performance-enhancing drugs and methods, risking their health, even their lives. Elite sports use whatever technology available to improve the performances (Pielke, 2016), increasing the athletes' risks for damaging their health. These risks go against the UN's sustainability goal #3 (“Good Health and Well-being”).

Politically, the sport system claims “Sport for all.” Still, there is a discrepancy between visions and reality. Sports recruits *unequally* regarding gender, race, age, class, income, and other socio-cultural distinctions. The critical question is why? Gils et al. (2021), in a forthcoming study, found that recruiting mentors to supervise coaches in elite sports was based on three distinctions. First, recruiters selected possible mentors from their network. Second, competence and trust were qualities the recruiters looked for, but they could not explicitly define these qualities when questioned. Third, the recruiters did not consider gender balance critical to meet the organization's demands and achieve the desired performances. Gils et al. interpreted these distinctions as an expression of *institutional logic*, a performance logic that permeated the whole elite sport system. This performance logic trumps the social-democratic ambitions of equality and the institutional commitment to follow this national policy. More generally, the performance logic outperforms sustainability goal #5 (“Gender Equality”).

Sport triggers emotions and *raptures*, in particular, elite sport and significant sports events. Flyvbjerg (2014) identified four sublimes behind the bidding process for mega-events. These explain stakeholders' involvements and raptures, such as engineers, politicians, entrepreneurs, and architects, as a promising alternative. I suggest a fifth sublime, the “interest group sublime”, the rapture that advocates of sporting practices get from constructing a building or arena which is much bigger than they had envisaged. This rapture was very apparent in the Oslo case (Tangen, 2021). Janis (1982) argued that *group-euphoria* tends to develop in temporary organizations in the form of buoyant optimism, a leader with great promise of hope, and a shared belief that the group could make “the future unlimited.” There are indications that euphoria has developed in the groupthink of intermediary and temporary groups applying for mega-events such as World Cups and bidding for Olympic Games, reducing the ability to make rational decisions. Solberg et al. (2018) and Jensen (2020) suggest that euphoria drove the decisions in the initial phase of applying for the UCI Road World Championships in Bergen 2017 and Oslo 2022, resulting in “irrational decision-making” (Heldal and Solberg, 2019). However, more research is needed to confirm these interesting theoretical concepts. Here, it is essential to emphasize how interests and raptures force local communities and local politicians to vote for events that threaten several UN's sustainability goals (#6 “Clean Water and Sanitation,” #8 “Decent Work and Economic Growth,” #12 “Responsible Consumption and Production,” #13 “Climate Action,” #14 “Life Below Water,” #15 “Life on Land,” #16 “Peace, Justice and Strong Institutions”). These threats also appear in other circumstances.

The professionalization and commercialization of modern sports imply a tremendous amount of money and wealth. This vast wealth is, for some inside the sport system, an opportunity for *easy money*. Critical journalism and scientific studies have exposed cheating, bribery, corruption, match-fixing, and doping in sports (Pielke, 2016). Athletes, coaches, team doctors, and sports executives have cheated and taken money to manipulate competitions. Such behavior threatens the core of the sport. Cobus de Swardt, Managing Director of Transparency International, puts it this way: there exist “… a culture of impunity at the top of sporting organizations that gives free rein to bribery and obscures financial black holes.” (de Swardt, 2016; p. xiii). When victories are purchased or manipulated, the reason for competition becomes futile. Accusations about corruption have also been raised against bidders for mega-events. They have paid decision-makers in the international sports federations to vote for their bids. Pielke (2016). considers sport faces a war against cheating and corruption. World Economic Forum warns that “Corruption has the potential to undermine the successful implementation of all 17 [SDG] goals^4^.” Without removing the flows of money in sports, these incidences will not disappear, and sustainability will be challenging to reach.

Sport's structural couplings to other systems, indicated in the examples above, influence all these systems' sustainability: the social subsystems, the ecological environment, and human biology and psychology. These relations made modern sport possible and significant but also dangerous in terms of sustainability. Let me very briefly indicate my points. Without *scientific knowledge* and different *technologies*, the improvement of performances would soon have reached a standstill. I wonder whether Bob Hays' 100-meter record at 9.91 seconds during the 1964 Olympics could have been improved very much without surface mounted starting blocks, synthetic surfaces on the tracks, better equipment, and performance-enhancing substances methods (both legal as well as illegal). This technological development holds for all other sports as well. The examples are endless. However, the consequences of the technologization of the sport system are alarming. Sport seems to turn all stones to get the edge (Pielke, 2016). The American social scientist John Hoberman argued years ago that the science of performance and the sport's dehumanization made sport “a gigantic biological experiment carried out on the human organism” (Hoberman, 1992). This technologization of sports threatens the UN's #3 “Good Health and Well-being.”

The spectacular and exemplary sports performances and contests became one of *modern mass media's* most exciting themes and topics, second only to the news. Therefore, the contemporary sport could ask for substantial sums of money to give mass media exclusive rights to broadcast the contests and events, such as the Olympic Games, the World Cups of different sports, the Tour de France, and the Super Bowl. This development enriches both sport and the mass media. Metaphorically speaking, these vast economic resources have worked like gasoline to the Olympic flame and its universal motto, *Citius–Altius–Fortius*.

Other businesses followed suit. *The sporting goods market* is a billion-dollar industry. In the United States alone, sporting goods generate more than 47 billion U.S. dollars annually. On the supply side, quoting (Andreff and Szymanski, 2006; p. 27), “the sports goods industry is an oligopoly dominated by a handful of transnational corporations.” They further claim that innovations have become crucial in this industry, partly because it helps athletes win or improve their performances, partly to make the sport more spectacular, partly to test new products and technologies, and partly to facilitate mass access and participation. This development is not sustainable regarding UN's sustainability goal #12 “Responsible Consumption and Production.”

Since the ancient policy of “*bread and circus*,” sport is still used politically. Today, sport is considered politically relevant for fostering democracy and voluntary public engagement. However, we observe that both democratic and authoritarian regimes use sport to *increase international recognition* and global power. In particular, bidding for and organizing Olympic Games and World Cups seems very tempting for large cities and nations. Despite the experiences that most such mega-events fail to give the expected outcomes, the bids are still coming. However, some possible bidders are alarmed by research showing that *mega-events overrun* with 100% consistency (Flyvbjerg and Stewart, 2012). Overruns in these games have historically been significantly more extensive than for other types of mega-projects. Such consequences do not advance sustainability in the sense of #11 “Sustainable Cities and Communities.”

As mentioned, the sport has become increasingly aware of its responsibility for the sustainability of the environment, particularly nature. However, the sport system still hesitates to deal appropriately with one major issue that influences the climate: *traveling*^5^ Local, regional, national, and global sports presuppose individual athletes and teams traveling to different places, competing for medals, diamonds, points, and fame in their pursuit for sporting omnipotence. Using cars, coaches, and airplanes—most using fossil energy—that pump carbon dioxide into the atmosphere transport the athletes, spectators, and supporters to the events. However, some awareness of this challenge exists. But will sport follow up on this crucial issue and reduce its carbon emission from traveling to almost nothing? I doubt that. Just let me give a few examples of how this traveling threatens UN sustainability goal #13 “Climate Action.”

In Agenda 2020 (Recommendation 4 and 5), the IOC *proclaimed* it would reduce its travel impact and offset its carbon emissions^6^. It is not clear whether this only goes for the IOC members and staff or includes all the athletes, support networks, supporters, spectators, and tourists who travel by the hundreds of thousands to host cities worldwide. FIFA has also produced sustainability documents regulating the FIFA World Cup, such as in 2014 (Brazil) and 2018 (Russia)^7^. Both organizations mention transportation and carbon management. However, it seems that this only covers transport within the host cities, not the transport of national teams and spectators worldwide. Why this forgetfulness?

In the “Introduction,” I mentioned the somewhat ironic solution to the carbon emission problem of transport of athletes by buying carbon offsets. The responsibility for carbon reduction is transferred to people in developing countries. They are expected to plant trees, making more effective cooking stoves or building wind farms. The complexity, morality and effectiveness of this “solution” have been heavily debated. The Guardian journalist and author George Monbiot consider this as a way of paying for the travelers sins^8^ However, airlines have offered voluntary carbon offsetting for over a decade, but <10% of air travelers purchase them (Ritchie et al., 2020). These authors suggest more research to understand carbon offsetting and sustainability's political and social context based on their findings. In this particular context, the athletes', sports organizations' and sport event hosts' values and opinions regarding sports traveling and carbon offsetting seem to an important research theme.

Another example of lack of ethical and sustainable consciousness is also expressed in sponsoring the national Norwegian ski jumping team. In an advert, the male and female athletes are lined up under the headline “The Sky is the Limit.” The sponsor is the Norwegian weapon manufacturer NAMMO. Norway is the 10th largest arms exporter, with 2.5 billion NOK revenue.^9^ The largest group of arms was “bombs, grenades and torpedoes,” with 1.1 billion NOK revenue. These weapons' severe effects on the three pillars of sustainability: environmental, economic and social, in the regions they were used, are not documented. However, one can easily imagine the effect weapons have on UN's #16 “Peace, Justice and Strong Institutions.” However, this sponsorship made it possible for the national ski jumping team to compete rather well and bring joy to their country's supporters. How should one weigh these outcomes against each other?

To sum up, I find it hard to believe that sport can be internal and external sustainable in modernity. One reason for this is sport's neglect and denial of the most significant challenge concerning sustainability: its own hypertrophic and pathological double-code. The idea of performance-enhancing and sporting omnipotence is so ingrained in today's elite sports that it may prove very difficult to change. This also apply to leisure sport, but in a lesser extent. Here too the performance code is operative, but in a weaker version. In both forms of sport, the transport of athletes and spectators are a major threat to sustainability. The second reason is the inevitable connections between sport and other subsystems in modern society. The exchange of support, resources, competencies, technologies, equipments, and performances uphold and increases the unsustainable impact of sports; metaphorically speaking: it is like pouring gas on the fire. However, these reasons are two sides of the same coin. The attributes of modern sports mirror the characteristics of modernity. “It is not muscle that wins. What wins is a certain idea of man and of the world, of man in the world …” (Barthes, 1961/2007; p 42). In the words of the Norwegian peace researcher Johan Galtung, one could argue: “Sport is a carrier of deep culture and structure.” (Galtung, 1982), carrying a message of being competitive in ranking nations, teams and individuals. The deep culture and structure of modern sports are, metaphorically speaking, a blueprint of modernity's DNA. This blueprint is the modern sport's blind spot. Sport cannot, and will not, see that its distinctiveness and operations are unsustainable. Instead, sport is fundamentally concerned about how it can operate according to its logic and double-code. And modern sport continually looks for collaborators to run faster, jump higher and be stronger.

Sport reproduces and sustains modernity's DNA in a more explicit and observable way than other social subsystems. This reproduction is sport's societal task, its function (Tangen, 1997). It displays and legitimizes the logic of progress and growth. Mirroring modernity's logic, its embedded contingency, sport paradoxically contributes to modernity and its demise. In this regard, the sport has a valid excuse for its unsustainable behavior; it acts on behalf of modern society. The blame for unsustainability is, therefore, on modern society. Unsustainability is a symptom of a society in crisis. As before in history, modernity produces its demise. Oswald Spengler once predicted, “*Der Untergang des Abendtlandes”* (or “the decline of the West”). As described above, Diamond (2005) and Turchin (2007) recently launched an even more depressing analysis of human societies' fates and empires' rise and fall. Is this where modern society is heading? Will modernity fall? Do we have to change or “leave” the modern society before sport becomes sustainable? Or is there another ways out for modernity, sport and sustainability? Could Harari's (2018) 21 lessons be applied with success? The answer to “Is there hope?,” depends on the possibility of changing the modern society. From my point of view, sequences of the DNA of modernity have to be manipulated to elicit change benefitting sustainability.

## The Dna of Modernity

Quite a few sociologists have tried to describe and explain modernity, including Marx, Comte, Simmel, Weber, Durkheim, Parsons, Elias, Giddens, Habermas, Galtung and Luhmann, to name a few. Without delving into the significance of their contributions, I will present a condensed description of some features that characterize modernity from Luhmann's system theoretical perspective. Luhmann's way of thinking are vital to deconstruct modernity and thereafter construct a solution to the consequences the individual, social and environmental consequences the modernity produces.

From an evolutionary perspective, society does not plan to change from, for example, a nomadic to a sedentary lifestyle. Sport does not plans to evolve from ritual to record, to paraphrase Guttmann (1978) and Luhmann (1997b). Evolution instead means waiting for *useable coincidences* or “mutants” (Luhmann, 1997b). Coincidences develop from deviance to differentiation, mostly without intention. From the end of the 16th to the middle of the 19th century, society increasingly reflected upon the contingent “nature” of things. Quite a few forms of societal actions (communications) became independent and autonomous to take care of specific problems (Luhmann, 1997b). Observing the issues of power, wealth, child-raising, justice, truth, and so forth as differences that had to be taken care of resulted in the emergence of autopoietic, or self-organizing, subsystems for each contingency.

Observing modernity, Luhmann (1998) claims that *contingency* is modern society's defining attribute. Embedded in every decision and action was the possibility that anything could be otherwise. “Anything is contingent that is neither necessary nor impossible” (p. 45). “Who is best?” and “How to improve?” is nothing but the defining contingencies in sport. When losing a competition, the losing athletes reflect upon “what could I have done differently?” Winning a game, the winning team asks themselves, “How could we improve to win again?” Decisions have to be made; performances have to be done, but with no guarantee of success. Contingencies determine society's development and the development of its subsystems like sport, politics, economy, science, mass media and so forth.

According to Luhmann, modern society “… experiences its future in the form of the risk of deciding.” (1998, p. 71). Every decision may result in undesirable results. There is no way to escape from contingency and risks. If a team participate to win, the team has to accept the possibility to lose. Choosing one method of performance-enhancing may prove wrong, but there are alternatives. The team must acknowledge that there is a “… difference between a judgement before and a judgement after the occurrence of loss.” (Luhmann, 1998; p. 71-72). Most decisions and efforts to improve performances will have sustainable consequences, environmental, economic, and social. However, today's decisions about the future are based on today's guesses about how the future will be. But the future may be different. We cannot know. The future is contingent.

This new, functionally differentiated society experimented with possible solutions to stabilize this social system's risks and contingency. Only those solutions that proved useful and solved the problem (that is, were functional) were accepted. Economy, politics, justice, science, and other areas became such functional subsystems. Each subsystem operated based on a distinct and unique difference, structured as a binary code. Moneyed/moneyless, power/powerless, legal/illegal and true/untrue became the code of society's economic, political, legal and scientific subsystem. As discussed above, the code of sport becomes win/lose with a secondary code of improving/decline.

As each subsystem followed its functional imperative, solving a particular problem, it became increasingly difficult for each subsystem to observe the distinctive feature of the society experiencing more and more issues regarding orientation and direction (Luhmann, 1997b). This increase in complexity resulted in an increasing non-transparency and a decreased possibility for steering. The society became decentred, with no privileged place from which to *steer*. No subsystem could, in principle, steer another, only irritate it. At best, each autopoietic subsystem could only steer itself, i.e., doing self-steering. Observing problems to solve is constructed internally. “The political system is in this respect no exception; politics too can only steer itself, and if the steering refers to the environment, then it is only to *its* environment” (Luhmann, 1997b; p. 46). The difficulty of steering the modern world to be more sustainable are evident.

Irritating other subsystems is only possible through what Luhmann conceptualizes as *structural coupling*, a mutual influence mechanism (1984). Each system decides its contact with its environment. It shields itself from countless influences while selecting a few relationships. When a particular company offers a given sum for being the primary sponsor for a specific sport, the sport in question can accept the offer but has to abstain from cooperation with other companies. Structural coupling allows the system to maintain its operational closure and autopoiesis, thereby developing the athletes' and the team's performances.

The functionally differentiated society *spread worldwide*. Other scholars have used terms like colonization, industrialization, and globalization to refer to this process. Luhmann claims that the functionally differentiated society has become a world society, where the functional subsystems dominate and operate paradoxically while being more independent and more dependent on each other. The local and regional peculiarities become in no small degree washed away, even if local variations exist.

The principle of functional differentiation also resulted in *increasing individualization*. The functionally differentiated society offered all people the possibility of inclusion (Luhmann, 1997b). The semantics of this observation was formulated as the human rights of freedom and equality. However, the regulation of the inclusion/exclusion principle was now entrusted to the functional subsystems. The political system itself determines political eligibility. The establishing and maintaining of the family was left to the family system. The economic system regulates participation according to wealth and income (Luhmann, 1995). The sport itself determines who is going to be an athlete or selected to the team. This positions the individual to freely choose whatever opportunity, lifestyle, or career he or she sees as suitable. However, it is crucial to notice that even if human rights secure equality, participation in sport is still not for all. Gender, class, ethnicity, religion still work as barriers to equal involvement and imply social unsustainability. In addition to increased possibilities of inclusion, the individualization process in modernity gave each individual the right to think, speak and act freely. This possibility poses a particular challenge to the implementation of political measures regarding sustainability (more on this later).

The encompassing contingency of modernity leads toward *hypertrophy*, an abnormal urge to grow (Luhmann, 1994). This urge characterizes all the subsystems and society's communication and operation at large. Subsystems are almost forced to grow but are themselves unable to control this growth. Each subsystem and the society at large increased in complexity. When this became apparent and observable about 1,800, the idea of “progress” was coined. Progress was positive and desirable, whereas the decline was not. This idea became the code that ordered all communication and operation in modernity. The sport experienced and coined this idea in its Olympic motto.

Contingency, expressed in the idea of progress, induced *self-reflective* processes within the modern society as a whole, in each of its subsystems and in each individual. Despite the contingent nature of modernity, or maybe a result of just this attribute, every subsystem tried to develop rational and efficient ways to develop itself, be it better policies, larger profits, more justice, truer truths, all manifestations of the DNA of modernity. This DNA has undoubtedly led to a better life for most of the world's populations. Paradoxically, this DNA is also responsible for all the problems related to steering, pollution, global warming, climate change, and so forth, putting us all in the situation to take sustainability very seriously. There are limits to growth in nature, modernity and sport, to paraphrase Meadows et al. (1972). In other words, the modern aims for progress and development threatens the balance of the ecological, social and individual systems. Could we hope for a solution to this self-inflicted threat? Could sport and society become sustainable given the autopoietic “nature” of both these social systems? Will the population in general and the sports followers in particular acknowledge the threat and act accordingly?

## Is There Hope?

The reader may experience this article as a rather pessimistic and gloomy view on the sport and its possibility of being sustainable. Hence, I will address one final question: *Is there hope?* Is it possible for the sport to be sustainable? It depends on how individuals, organizations and social systems respond to the six significant issues or problems discussed above. I draw the following seven conclusions:

First, as indicated above, the individualization process in modernity gave each individual the possibility to think, speak, decide and act freely according to how each individual observes the surrounding world. However, there is no guarantee that billions of people will agree to the available information and knowledge concerning sustainability and act accordingly. Psychologists, economists, and philosophers have been studying individual decision-making, using concepts like *wishful thinking* (Neuman et al., 2014), *willful ignorance* and *self*-*deception* (Alicke, 2017; Wieland, 2017). In the terms of Kahneman (2011), human decision making is flawed due to *heuristic* and *biased* cognitive processes. These psychological traits are essential sources for socially harmful behavior (Grossman and van der Weele, 2016). In this particular context, willful ignorance may shape how athletes, sports leaders, sports enthusiasts, people in general observe the scientific information about climate change, pollution, social inequality etc. If they consider the information and knowledge exaggerated and probably false, they are in a state of willful ignorance and will be unwilling to accept changes toward more sustainable sport and society. Given the autopoietic nature of human consciousness (Luhmann, 1984), it is challenging to convince individuals to think and respond “from outside.” Each consciousness produces its own “world view” from its observations of its surroundings to “survive”, which might results in grave societal consequences.

Second, my rather superficial reading of current exemplary research indicates that sport does a lot to be sustainable and has had some success with the efforts. However, the sport, the current research and the public discussions seem to have *little awareness* of modern sports' embedded logic and how this logic is defined and strengthened in modernity and severely impacts sustainability. Our knowledge is insufficient and flawed. So are our actions to solve this threat. The lack of awareness and knowledge result in a too optimistic conclusion about whether the sport is sustainable (wishful thinking). Suppose we increase the understanding of the dangers of modern sport's double-code and acknowledge its complex entanglement with the surrounding society, civilization and ecological environment. In that case there may be hope to counteract the dramatic consequences.

Third, my reading also revealed that different meanings of a concept lead to various answers and diversified actions and policies. For example, the sport system may believe in *sustainable development* and decide to follow the guidelines for sustainable efforts: reduce consumption, increase recycling, measure and report according to sustainability standards, better the Earth's carrying capacity, reduce the human impact on biodiversity, etc. The sport system may also subscribe to the idea of sustainable development by using fully electric transportation systems like electric bus, electric train, and the hope that electric airplanes soon will be a reality. The sport system may endorse a belief in sustainable development by enhancing performances by all sustainable means. However, the two terms, sustainability and development, seem to contradict each other (Barker et al., 2014). In society, sustainability and development could not simultaneously be achieved. To unite them is comparable to the task of squaring the circle (Robinson, 2004). Continually improving performances should not be the goal if the sport aims to be environmental, economic and socially sustainable. Quite the opposite seems to be necessary.

Fourth, to achieve sustainability is to go for a *recession* of sport and a decline in the performances if we follow this kind of conceptualization. Is that possible considering the current performance logic of sport? Indirectly Loland (2006) points to the logic of sport in his analysis of sport and sustainability. He suggests that if sport aims to be sustainable, it has to reform or abstain from specialization and record mania. This reform implies a move back to the basics, i.e., discard the secondary code of improving the performances. We could, as an *ethical recess*, go back to the amateur ethos of the English Gentleman's sports when athletes “competed under a prevailing attitude of fairness and sportsmanship, and serious and extended practicing or training for a sport was considered synonymous with cheating” (Sage, 2010; p. 43).

Or similarly, a *cultural recess* may be a solution. We could abandon the specialization and record mania by looking at other cultures and their sports, such as the Tikopian dart game described by Raymond Firth in the 1930s (Firth, 1930). In this game, winning was essential and losing shamefully. Nevertheless, it was a big occasion for the whole community to celebrate. The arena was dug out and made ready for the game. Interestingly, after the game, the winners would go off to their orchards to pluck coconuts and distribute them to the losers. Both teams would then sit down to drink, eat, and refresh themselves. According to Firth, this was an example of “*winners pay*.” This type of contest could also be an example of a sustainable sport. It is about winning, but not about continually improving performances. Like the Gentleman's sports, it lacked the secondary code of modern sport, improvement and was probably sustainable despite the temporary deforestation building the sports arena.

Breivik (2019) suggests another form of recess. He describes changing sport based on Arne Naess's ecological philosophy. This change implies a form of a *recess of values*. He claims, “… one could change sports through a series of steps from shallow to more deep ecological versions.” (p. 79). One step is to minimize the use of resources and energy but inside the given sports patterns. A second step is to support and sponsor sports and sport forms that use renewable resources and simple means. The third step involves, wherever possible, sporting activities in natural settings and simple means should be developed. The fourth step addresses the personal level: “one should try to become wise sportspersons who can realize deep ecological concerns through spontaneous play, by touching the Earth lightly and with an ideal of richness in ends and simpleness in means.” (p. 79).

Fifth, Loland's and Breivik's proposals only addres*s* the symptoms, not the core of the matter. The reason for sport's unsustainability is, in my opinion, the contingent, hypertrophic and pathological code of sport—mirroring the DNA of modernity. Both sport and society need to change. Even though the analysis of societies and civilizations of Diamond, Turchin and Harari show the importance of looking at the question of sustainability from a macro-perspective, I do not find their analysis quite compelling either. I accept their statements that modernity as we know it is in a deep crisis, as other human civilizations have been before (Diamond, 2003, 2005; Turchin, 2007; Harari, 2018). However, despite their books' overwhelming details and scope, I find their solutions to this crisis surprisingly simplistic. After addressing 12 sets of sustainability problems, resulting from different kinds of disastrous decisions humans have made through history, Diamond (2005) is still cautiously optimistic. He sees a possibility for modernity not to collapse in the globalized worlds interconnectedness. In particular, the television and other mass media gives an “… opportunity to learn from the mistakes of distant peoples and past peoples.” (p. 525). Turchin suggests a similar solution. People could be mobilized through the Internet and especially mobile phones to enhancing the *asabiya* (Turchin, 2007)—the capacity for collective action for sustainability. Turchin seems to see asabiya, or social capital in Putnam's terms, as a solution to save and reshape modernity. Harari (2018) falls back to individual solutions. He suggests meditating 2 h a day. That may help persons who feel helpless and depressed by the alarming and threatening news about global warming and increased water and air pollution. In a social, political and economic context, Harari's solution appears individualistic, superficial and unfounded. On the other hand, maybe there is no other solution to the sustainability challenge than resign, meditate and survive individually?

Sixth, is it possible for sport to change its double-code and make a recess in a contingent society where the autopoietic code of progress reigns? Is it possible to plan a change in sport and society that give hope for a sustainable world? Is it possible to pull the population in general, and sports enthusiasts, athletes and leaders in particular, out of their wishful thinking, willful ignorance and self-deception and start on a more sustainable path of living? To change, or more precisely replace, peoples' thinking and the unsustainable double-code of sport, we have to change the society we call modernity. Sport is nothing but a blueprint of the surrounding society, carrying its DNA. However, as I mentioned above, societies do not change according to plans. The evolution of new societies means waiting for *useable coincidences* (Luhmann, 1997b). Coincidences develop from deviance to differentiation, mostly without intention. Ironically, it may be possible that sustainability is the useable coincidence that triggers the development of a new society and its subsystems like sport. The alarming IPCC Climate Change Report indicate a “Code Red for Humanity.” This report may spur thorough self-reflection at all levels in society, within its subsystems and population. from this One could speculate whether the self-reflections will bring forward new ways of coping with the sustainability challenge, find new ways of reaching collective binding decisions that benefits sustainability, or invent new ways of producing the required energy to maintain a sustainable standard of living and so forth? However, it seems unlikely that this takes place before it is too late. To me, only an extensive social, cultural, economic recession is what it takes to reach global sustainability.

Observing modernity in general and the ecological future in particular, Luhmann wrote: “By now, one thing is clear: evolution has always been, to a great extent, self-destructive, both in the short and long term. … almost all cultures that have affected human life have disappeared. … Cultural forms that are self-evident today and the “world” of today's society will meet a similar fate. No one can seriously doubt this” (Luhmann, 1998; p. 75). Luhmann finds it rather probable that humankind as a life form will someday disappear. He sees three scenarios. (1) Humans may replace themselves with genetically superior humanoid life forms. (2) It is also possible that humans will eradicate themselves through human-made catastrophes. (3) Or maybe in the future, people will “… destroy the common technological devices we take for granted to such an extent that only a very elementary form of survival will remain possible.” (p. 75). That sport should have a future, or more precisely existence, seems unlikely in any of these scenarios. On the other hand, different sports have been a part of all the civilizations we know of. So, in the long run there may be hope for some sport practices in the sustainable society and civilization that eventually emerge. This rather depressing conclusion may horrify some, provoke others, and make a few to look for another theory.

To me, Luhmann's theory gives the best answer to whether sport can be sustainable. The solution should invoke a thorough self-reflection and discussion of this issue inside the sport system. The usable coincidence to bring forward changes in the sport's double-code is the same as for the society, namely the knowledge distilled in the IPCC report, indicating “Code Red for Humanity.” Each sport should reflect upon the double-code of winning and improvement and how each sport's uniqueness and distinctive character impact different sustainability forms. Given the analysis above, they could, or should, not reach any other conclusion than sport has to recess, going back to basic forms of sports. Whether that is possible depends on how modernity similarly and synchronously will reflect upon its contingent and pathological “nature” and act accordingly. As implied above, modern society has to change or replace itself into a more simple but global, low-tech based society where playful, physical contests may take place locally and be sustainable. This form of society is the only solution from my perspective; a world society that operates and governs locally and globally from the distinction or binary code of “sustainable/unsustainable,” not the statal power/powerless code that rules modern societies.

Seventh, since society cannot plan and steer for the future, society, sport, and individuals have to hope for usable coincidences. Suppose the alarm “Code Red for Humanity” is observed as a usable coincidence. In that case, it might induce self-reflection and communicative responses in individuals, organizations and social sub-systems, and produce vital changes benefitting sustainability. However, we have to wait and see what kind of sport generates from the manipulated DNA of this eventually new, global, sustainable society, if at all. When waiting for the future to unfold, and reflecting and discussing consequences and possibilities, I suggest we, in addition to hope and meditate, act according to the precautionary principle—and listen to Einstein: “We cannot solve our problems with the same thinking we used when we created them.”

## Data Availability Statement

The original contributions presented in the study are included in the article/supplementary material, further inquiries can be directed to the corresponding author.

## Author Contributions

The author confirms being the sole contributor of this work and has approved it for publication.

## Conflict of Interest

The author declares that the research was conducted in the absence of any commercial or financial relationships that could be construed as a potential conflict of interest.

## Publisher's Note

All claims expressed in this article are solely those of the authors and do not necessarily represent those of their affiliated organizations, or those of the publisher, the editors and the reviewers. Any product that may be evaluated in this article, or claim that may be made by its manufacturer, is not guaranteed or endorsed by the publisher.

## Footnotes

^1^This article is a revised and extended version of my key note at EASS Conference “Sport and the Environment,” June 3-6, Bø I Telemark, Norway.

^2^https://chooose.today/partner/.

^3^http://www.anecologyofmind.com/gregorybateson.html (read 21.01.2021).

^4^https://www.weforum.org/agenda/2019/09/serious-about-sustainability-get-serious-about-corruption/ (read, January 2020).

^5^Transport with large cars and long flight (business class) are the two largest contributors to carbon intensity http://shrinkthatfootprint.com/shrink-your-travel-footprint.

^6^https://stillmed.olympic.org/Documents/Olympic_Agenda_2020/Olympic_Agenda_2020-20-20_Recommendations-ENG.pdf (read, April 2019).

^7^https://resources.fifa.com/image/upload/sustainability-report-of-the-2014-fifa-world-cup-2509269.pdf?cloudid=educsd2hgasief3yeoyt (read, April 2019).

^8^https://www.theguardian.com/environment/2006/oct/18/green.guardiansocietysupplement.

^9^https://www.ssb.no/utenriksokonomi/artikler-og-publikasjoner/okt-eksport-av-vapen-i-2015?tabell=252747 (read, January 2021).
